# Interpretable machine learning analysis of the impact of tranexamic acid combined with antibiotics on renal function and its risk factors in hip replacement surgeries

**DOI:** 10.3389/fsurg.2026.1869935

**Published:** 2026-08-21

**Authors:** Lijuan Xu, Xiaowang Lan, Yafang Jiang, Haoyu Wang, Jiangbao Xu, Bingsheng Liu

**Affiliations:** 1 Zhejiang Chinese Medical University, Hangzhou, China; 2 Department of Orthopedics, Quzhou People's Hospital, Quzhou Affiliated Hospital, Wenzhou Medical University Hospital, Quzhou, China

**Keywords:** LightGBM, machine learning, RF, Scr, TXA

## Abstract

**Background:**

Early prediction of renal function changes in patients undergoing hip replacement surgery enables timely intervention for acute kidney injury and acute renal failure, which in turn improves treatment outcomes. This study aimed to develop and validate an interpretable machine learning model that uses real-world clinical parameters to predict postoperative renal function changes. The model incorporates tranexamic acid (TXA) and antibiotics as key predictive factors, examines their combined effect on renal function, and identifies associated risk factors.

**Methods:**

This observational study was conducted from January 2022 to June 2024 at Quzhou Affiliated Hospital of Wenzhou Medical University. After data preprocessing, the full dataset was randomly split into a training cohort and an internal validation cohort at a 7:3 ratio. We performed feature selection via support vector machine-recursive feature elimination (SVM-RFE) to identify the most clinically relevant predictive variables. Changes in serum creatinine (Scr) levels were defined as the primary outcome variable. We evaluated the predictive performance of four machine learning models using multiple standard assessment metrics. Feature importance was calculated for each candidate model, and the top-performing model was further interpreted using SHAP and LIME algorithms.

**Results:**

In both the training and validation cohorts, the LightGBM model outperformed RF, GBDT, and XGBoost in predicting Scr changes. SHAP analysis showed that the top five features contributing to Scr prediction in the LightGBM model were D-dimer difference, C-reactive protein (CRP) level, sodium difference, hemoglobin difference, and antibiotic use. DCA analysis indicated that all these models delivered superior predictive performance compared to traditional methods.

**Conclusions:**

The LightGBM model demonstrated higher AUC values in predicting Scr changes. TXA dosage was not identified as a top predictor of postoperative acute kidney injury in our models; however, antibiotic dosage was correlated with renal function outcomes. Our study identified and validated critical risk factors for postoperative renal impairment, including elevated CRP levels, sodium fluctuations, intraoperative blood loss, and transfusion volume. These findings have significant clinical implications for perioperative management, monitoring protocols, and risk stratification.

## Introduction

Acute kidney injury (AKI) is a clinical syndrome characterized by a rapid decline in renal function over a short period of time. The incidence of postoperative AKI among surgical patients ranges from 6% to 27%, and particularly high rates are observed in elderly patients undergoing hip arthroplasty, where the incidence reaches 16%–24.44% (1–5). This complication can readily progress to acute renal failure, leading to severe adverse clinical outcomes, including increased disability and mortality rates, as well as reduced patient quality of life (6–9). This substantial clinical burden imposes significant physiological, psychological, and socioeconomic pressures on both patients and healthcare systems (10).

The underlying pathophysiological mechanisms of this disease are typically associated with synergistic nephrotoxicity resulting from the concurrent use of hemostatic agents [e.g., tranexamic acid (TXA)] and antibiotics during the perioperative period. This combination therapy is widely employed in major orthopedic surgeries, as these procedures are frequently associated with significant blood loss and surgical trauma (11, 12). This context underscores the urgent need for rigorous research to elucidate the impact of TXA-antibiotic combinations on renal function and to identify risk factors for acute kidney injury (AKI) during orthopedic procedures.

In contemporary clinical practice, machine learning applications primarily span five domains: (1) medical imaging-assisted diagnostic systems (13, 14); (2) disease risk stratification models (15); (3) personalized treatment optimization strategies (16); (4) virtual drug screening platforms (17); and (5) intelligent hospital management systems (18).

Observational real-world studies offer unique advantages in capturing clinical heterogeneity, such as diverse patient demographic characteristics and treatment adherence patterns (19) These approaches enable integrated analysis of multidimensional clinical data encompassing patient characteristics, treatment regimens, and prognostic outcomes, thereby facilitating the development of intelligent diagnostic models for precise risk stratification and individualized outcome prediction. To date, no study has utilized real-world clinical data combined with machine learning methods to establish a risk prediction model for AKI associated with TXA-antibiotic combination therapy following hip arthroplasty.

Therefore, this study aims to develop a risk prediction model for this complication based on real-world clinical data using machine learning algorithms, and to validate and interpret the model's predictive performance, thereby providing decision support for preoperative identification of high-risk patients and optimization of perioperative medication regimens. Building on this framework, we conducted a retrospective analysis of real-world clinical data employing interpretable machine learning algorithms to achieve two primary objectives: (1) to evaluate the impact of concurrent use of tranexamic acid (TXA) and antibiotics following major orthopedic surgeries on renal function parameters (serum creatinine); (2) to systematically identify and validate intervenable clinical risk factors for perioperative acute kidney injury.

## Methods

This study adhered to the Guidelines for Reporting Updates of Clinical Predictive Models Based on Regression or Machine Learning Methods(TRIPOD + AI, Box 1).

Box 1 TRIPOD+AI checklist.

Glossary of terms used in TRIPOD+AI

The definitions and descriptions given below relate to the specific context of the TRIPOD+AI* guideline; they do not necessarily apply to other areas of research.

Artificial intelligence

Field of computer science that focuses on developing models and algorithms capable of performing tasks that typically require human intelligence.

Calibration

Agreement between observed outcomes and estimated values from the model. Calibration is best assessed graphically with a plot of the estimated values on the x axis and observed values on the y axis, with a smoothed flexible calibration curve in the individual data.               Yes

Class imbalance

When the frequency of individuals with and without the outcome event is unequal.           Yes

Care pathway

Structured and coordinated plan of care for managing a specific health condition or dealing with a patient’s healthcare needs throughout their healthcare journey.     Yes

Discrimination

How well the predictions from the model differentiate between individuals with and without the outcome. Discrimination is typically quantified by the c statistic (sometimes referred to as the area under the curve (AUC) or area under the receiver operating characteristics (AUROC)) for binary outcomes, and the c index for time-to-event outcomes.    Yes

Evaluation or test data

Data used to estimate the performance of a prediction model, sometimes referred to as test data or validation data.† Evaluation data should be distinct from the data used to train the model, tune hyperparameters, or do model selection, such that there is no overlap in participants between the training and evaluation data. Evaluation data should be representative of the population in whom the model is to be used.       Yes

Fairness

Property of prediction models that do not discriminate against individuals or groups of individuals based on attributes such as age, race/ethnicity, sex/gender, or socioeconomic status.    Yes

Hyperparameters

Values that control the model development or learning process.     Yes

Hyperparameter tuning

Finding the best (hyper)parameter settings for a particular model building strategy.     Yes

Internal validation

Evaluating the performance of a prediction model on the same population on which the model was developed (eg, train test split, cross validation, or bootstrapping).     Yes

Machine learning

A subfield of artificial intelligence that focuses on developing models capable of learning and making predictions or decisions from data, without being explicitly programmed.     Yes

Model evaluation

Evaluating predictive accuracy of a model by estimating model discrimination (eg, c statistic), model calibration (eg, calibration plot, calibration slope), and clinical utility (eg, decision curve analysis). This process is referred to as evaluating a prediction model.    Yes. Add New calibration curve

Outcome

Diagnostic or prognostic event that is being predicted. In machine learning, this event is often referred to as the target value, response variable, or label.       Yes

Predictor

Characteristic that can be measured or attributed at an individual level (eg, age, systolic blood pressure, sex, disease stage, radiomics features) or group level (eg, country). It is also often referred to as an input, feature, independent variable, or covariate.     Yes

Training or development data

Data used to train or develop a prediction model. The training data are ideally representative of the population in whom the model is to be used.

*TRIPOD, Transparent Reporting of a multivariable prediction model for Individual Prognosis Or Diagnosis; AI, artificial intelligence.    Yes

†Validation data often has different meanings. For example, in machine learning studies, validation data can refer to data used for parameter tuning or data used to evaluate model performance (often referred to as external validation). To avoid any ambiguity, we refer to data used to evaluate model performance as evaluation data.     Yes

### Data source

This study was conducted at the Orthopedic Center of Quzhou Affiliated Hospital of Wenzhou Medical University from January 2022 to June 2024. The study protocol was approved by the Hospital's Institutional Review Board (Reference number: LW2024–160) in accordance with the principles of the Declaration of Helsinki. Data were retrospectively collected from January to June 2024, and investigators had no access to personally identifiable information throughout the study period.

Inclusion criteria were: (1) undergoing major orthopedic procedures with intraoperative administration of TXA and antibiotics; (2) availability of complete perioperative documentation. Exclusion criteria were: (1) in-hospital mortality, voluntary discharge against medical advice, or incomplete clinical documentation; (2) TXA or antibiotic administration unrelated to the surgical procedure.

To ensure data quality and consistency, all clinical data were obtained solely from the institutional electronic medical record (EMR) system. Strict adherence to pre-established eligibility criteria was maintained throughout the participant selection process. Data collection and validation were implemented through a dual-entry verification protocol, with independent cross-checking completed by two researchers.

### Feature selection methodology

The study variables were rigorously selected through a multidimensional framework incorporating: (1) a comprehensive literature review of renal function determinants (20), and (2) expert input from senior clinicians (≥15 years of experience). The final feature set encompassed five domains:Demographic characteristics: including age, sex, body mass index, and admission type; Medical history: including hypertension, diabetes mellitus, coronary heart disease, smoking history, alcohol use, and prior treatments; Surgical parameters: including anesthesia method, operative duration, intraoperative blood loss, transfusion volume, TXA dosage, and antibiotic administration (vancomycin and other agents); Laboratory parameters: including preoperative and postoperative measurements of blood glucose, white blood cell count, neutrophil count, glycated hemoglobin, and high-sensitivity C-reactive protein (hs-CRP); Renal function indicators: changes in serum creatinine (Scr) measured within two days preoperatively and three days postoperatively.

### Data preprocessing

To enhance statistical power and minimize potential bias, continuous variables with missing data were handled using multiple imputation methods. Missing values were identified through Tukey's fences method and subsequently addressed by interpolation from adjacent values.

The processed dataset was then randomly split into a training set and a validation set at a 7:3 ratio, with stratified sampling retained. Serum creatinine (Scr) levels were designated as the outcome measure. According to KDIGO standards, the Scr outcome is defined as meeting any one of the following criteria: a postoperative Scr level ≥ 26.52 µmol/L (0.3 mg/dL) higher than the baseline level within 48 h postoperatively.

### Feature selection

To avoid suboptimal feature selection, we implemented a rigorous variable selection process using the training cohort to identify the most relevant predictors for model construction. First, pairwise Pearson correlation analysis was performed to evaluate collinearity among clinical variables. Collinearity—defined as a high correlation (*r* > 0.8) between two or more predictors—compromises the accurate assessment of each variable's individual contribution to the outcome. We therefore excluded the more readily obtainable variable from each correlated pair.

Next, the support vector machine–recursive feature elimination (SVM-RFE) algorithm was applied for further feature refinement. This method iteratively reduces the feature set, evaluates model performance, and identifies an optimal subset of predictors. We mandatorily retained antibiotic doses (including vancomycin and other antibiotics) and TXA dosage in the variable set (21).

### Model development and validation

Serum creatinine (Scr) after surgery were defined as outcome measures. We employed four ensemble learning algorithms—eXtreme Gradient Boosting (XGBoost), Random Forest (RF), Light Gradient Boosting Machine (LightGBM), and Gradient Boosted Decision Trees (GBDT)—to predict postoperative Scr changes. All models used the same set of clinical predictors.

Hyperparameter optimization was conducted via grid search and randomized search strategies on the training cohort. Model performance was evaluated using comprehensive metrics including the area under the receiver operating characteristic curve (AUC-ROC), area under the precision-recall curve (AUC-PR), F1 score, precision, recall, sensitivity, and specificity, calibration plots and Brier scores.

For model interpretation, we implemented two explainable AI techniques: Shapley Additive Explanations (SHAP) for global feature importance analysis and Local Interpretable Model-Agnostic Explanations (LIME) for instance-level interpretation. This dual approach provided interpretable insights with both global consistency and local accuracy, thereby enhancing the transparency of the optimal model's decision-making process.

To address potential selection bias due to limited sample size, we implemented rigorous 5-fold cross-validation during model development, ensuring the robustness and generalizability of our findings (21).

### Hyperparameters tuning

#### XGBoost

**Figure 1:**
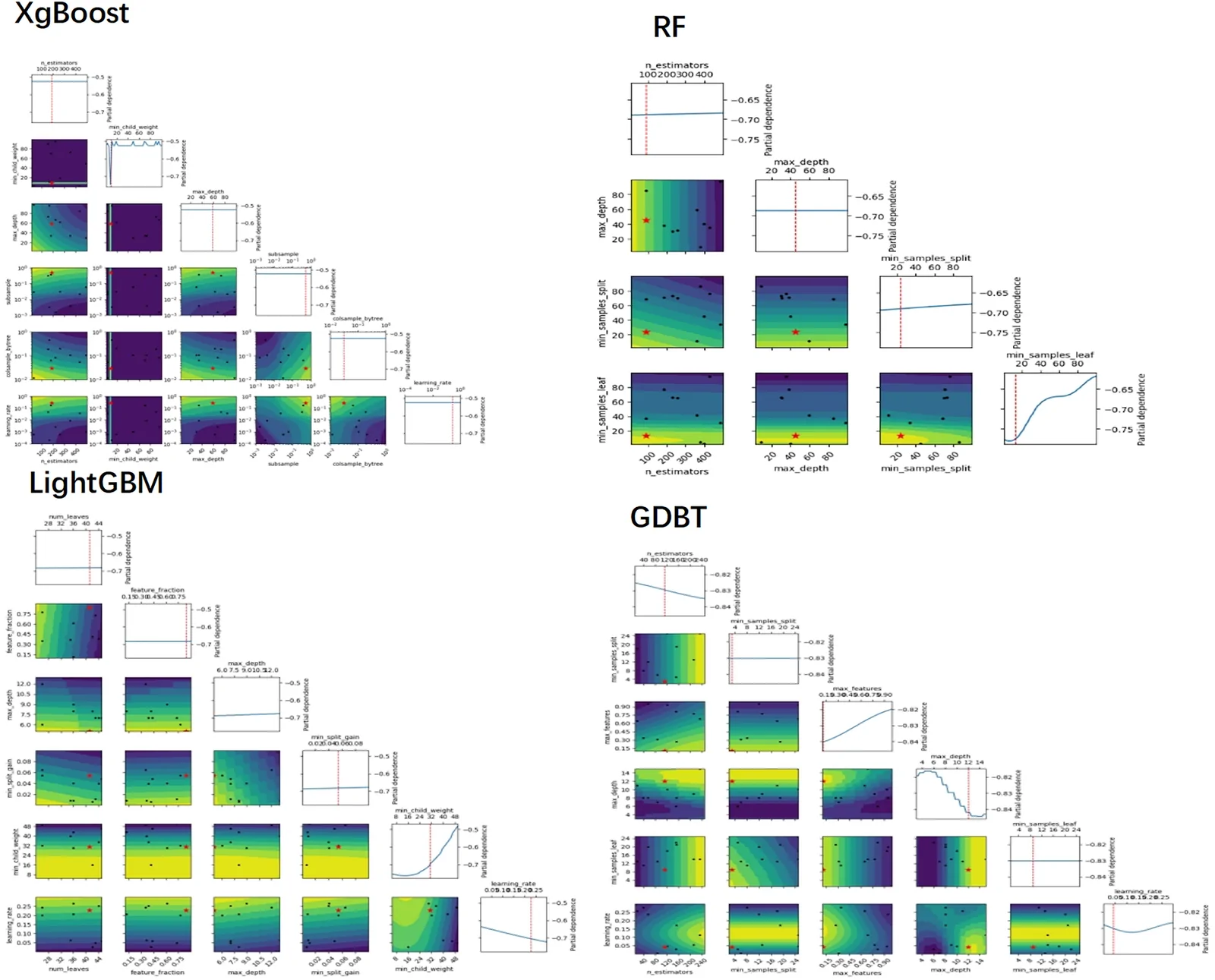
Data set selection plots.

XGBoost is a highly efficient ensemble learning method based on gradient boosting that integrates multiple weak learners, typically decision trees, to enhance predictive accuracy and robustness. Model tuning was performed using the XGBoost package with 5-fold cross-validation to determine optimal hyperparameters, as shown in Figure 1.

#### RF

Random Forest employs an ensemble of decision trees trained via bootstrapping to improve generalization and reduce overfitting. The optimal number of trees was selected through 5-fold cross-validation using the “randomForest” package, as illustrated in Figure 1.

#### LightGBM

LightGBM is a gradient boosting framework designed for high efficiency and scalability, utilizing histogram-based algorithms for accelerated training and lower memory usage. Hyperparameter tuning was carried out via 5-fold cross-validation with the LightGBM package, as depicted in Figure 1.

#### GBDT

Gradient Boosting Decision Trees (GBDT) is an iterative ensemble method that optimizes model performance by sequentially fitting trees to residual errors following the negative gradient direction. It is widely used for its flexibility and strong predictive performance on complex datasets. Model optimization was conducted using the GBDT package and 5-fold cross-validation, with results illustrated in Figure 1.

### Statistical analysis

Preliminary data assessment included normality testing using the Kolmogorov–Smirnov test with Lilliefors correction. Continuous variables were analyzed according to their distribution: normally distributed variables were compared using an independent samples *t*-test and are expressed as mean ± standard deviation (SD); non-normally distributed variables were analyzed using the Mann–Whitney U test and are reported as median [interquartile range (IQR)]. Categorical variables were analyzed using contingency table analysis: Pearson's *χ*^2^ test was applied when all expected frequencies were ≥5, and Fisher's exact test was used for cells with expected counts <5. Results for categorical variables are presented as *n* (%).

**Figure 2:**
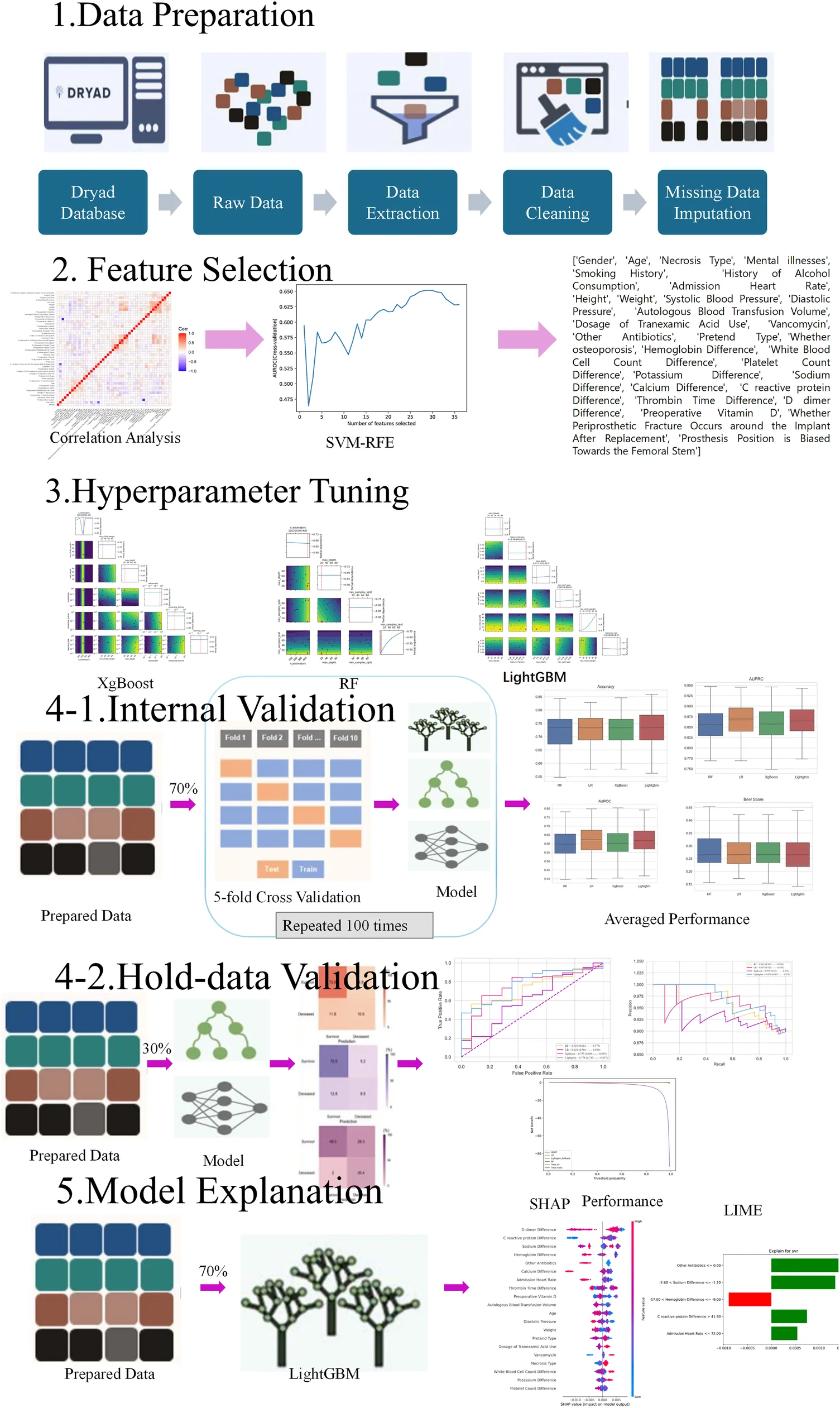
Flow chart.

Model discrimination was compared using DeLong's test for paired receiver operating characteristic (ROC) curves, with 95% confidence intervals (CIs) estimated via 2,000 stratified bootstrap resamples. Decision curve analysis (DCA) was used to assess the net clinical benefit across risk thresholds. To ensure model stability, we maintained a minimum of 10 events per predictor variable throughout the analysis. All statistical analyses were performed using R (version 4.2.2; R Foundation for Statistical Computing) and Python (version 3.9.0; Python Software Foundation). The entire process is shown in the Figure 2.

## Results

### Patient characteristics

The final dataset comprised a total of 459 patients who underwent elective hip arthroplasty. The cohort was randomly stratified by key covariates into a training set (*N* = 321) and a validation set (*N* = 138). No statistically significant differences were observed in baseline demographic, clinical, or surgical characteristics between the two groups. Detailed baseline characteristics are summarized in Tables 1, 2.

**Table 1:** Summary table of categorized data.

Characteristics	Totals (*n* = 459)	Training cohort (*n* = 321)	Validation cohort (*n* = 138)	*p* value
Gender	0.48	0.50	0.41	0.09
Necrosis type	0.37	0.37	0.37	1.00
Mental illnesses	0.03	0.03	0.02	0.80
Smoking history	0.18	0.19	0.17	0.64
History of alcohol consumption	0.18	0.20	0.14	0.24
Other antibiotics	0.24	0.26	0.19	0.12
Pretend type	0.00	0.00	0.01	1.00
Whether osteoporosis	0.34	0.34	0.33	1.00
Periprosthetic fracture	0.07	0.06	0.08	0.63
Prosthesis position	0.21	0.20	0.22	0.63

**Table 2:** Summary table of continuous variables data.

Characteristics	Totals(median)	Median[1/4, 3/4]	Train(median)	Train-Median[1/4, 3/4]	Validation(median)	Validation-Median[1/4, 3/4]	*p* value
Admission Heart Rate	77	77 [72, 84]	78	78 [72, 84]	75	75 [70, 83]	0.18
Height	160	160 [155, 165]	160	160 [156, 166]	159	159 [153, 165]	0.82
Weight	60	60 [52.3, 67.8]	60	60 [53, 680]	59.5	59.5 [51.4, 67.48]	0.33
Systolic Blood Pressure	139	139 [127, 153]	140	140 [127, 154]	137	137 [125, 151]	0.39
Diastolic Pressure	78	78 [71, 85]	78	78 [72, 85]	76	76 [69.25, 84]	0.14
Autologous Blood Transfusion Volume	200	200 [0, 250]	200	200 [0, 250]	200	200 [0, 250]	0.77
Dosage of Tranexamic Acid Use	2	2 [2, 2.5]	2	2 [2, 2.5]	2	2 [2, 2.5]	0.39
Vancomycin	1,000	1,000 [1,000, 1,000]	1,000	1,000 [1,000, 1,000]	1,000	1,000.0 [1,000, 1,000]	0.96
Hemoglobin Difference	−18	−18 [−25, −10]	−17	−17 [−25, −9]	−18	−18 [−24.75, −12]	0.33
White Blood Cell Count Difference	3.4	3.4 [0.6, 5.9]	3.1	3.1 [0.4, 5.8]	4.2	4.2 [1.2, 6.3]	0.07
Platelet Count Difference	−17	−17 [−38, 7.5]	−17	−17 [−39, 11]	−17	−17 [−35, 4.75]	0.74
Potassium Difference	3.91	3.91 [3.67, 4.16]	3.91	3.91 [3.67, 4.16]	3.92	3.92 [3.7, 4.21]	0.22
Sodium Difference	−1.1	−1.1 [−2.65, 0.5]	−1.1	−1.1 [−2.6, 0.5]	−1.05	−1.05 [−2.9, 0.5]	0.69
Calcium Difference	−0.18	−0.18 [−0.25, −0.08]	−0.18	−0.18 [−0.26, −0.08]	−0.17	−0.17 [−0.25, −0.06]	0.59
C reactive protein Difference	26.8	26.8 [12.5, 40.41]	27.26	27.26 [12.5, 41.9]	26.255	26.26 [11.6, 38.68]	0.59
Thrombin Time Difference	−0.3	−0.3 [−1.4, 0.6]	−0.3	−0.3 [−1.3, 0.7]	−0.4	−0.4 [−1.48, 0.6]	0.31
D dimer Difference	1.09	1.09 [−0.04, 1.94]	1.02	1.02 [0.0, 1.93]	1.21	1.21 [−0.49, 1.94]	0.96
Preoperative Vitamin D	25.11	25.11 [18.77, 33.115]	25.11	25.11 [18.94, 33.14]	25.06	25.06 [18.33, 32.49]	0.84

### Feature selection

**Figure 3:**
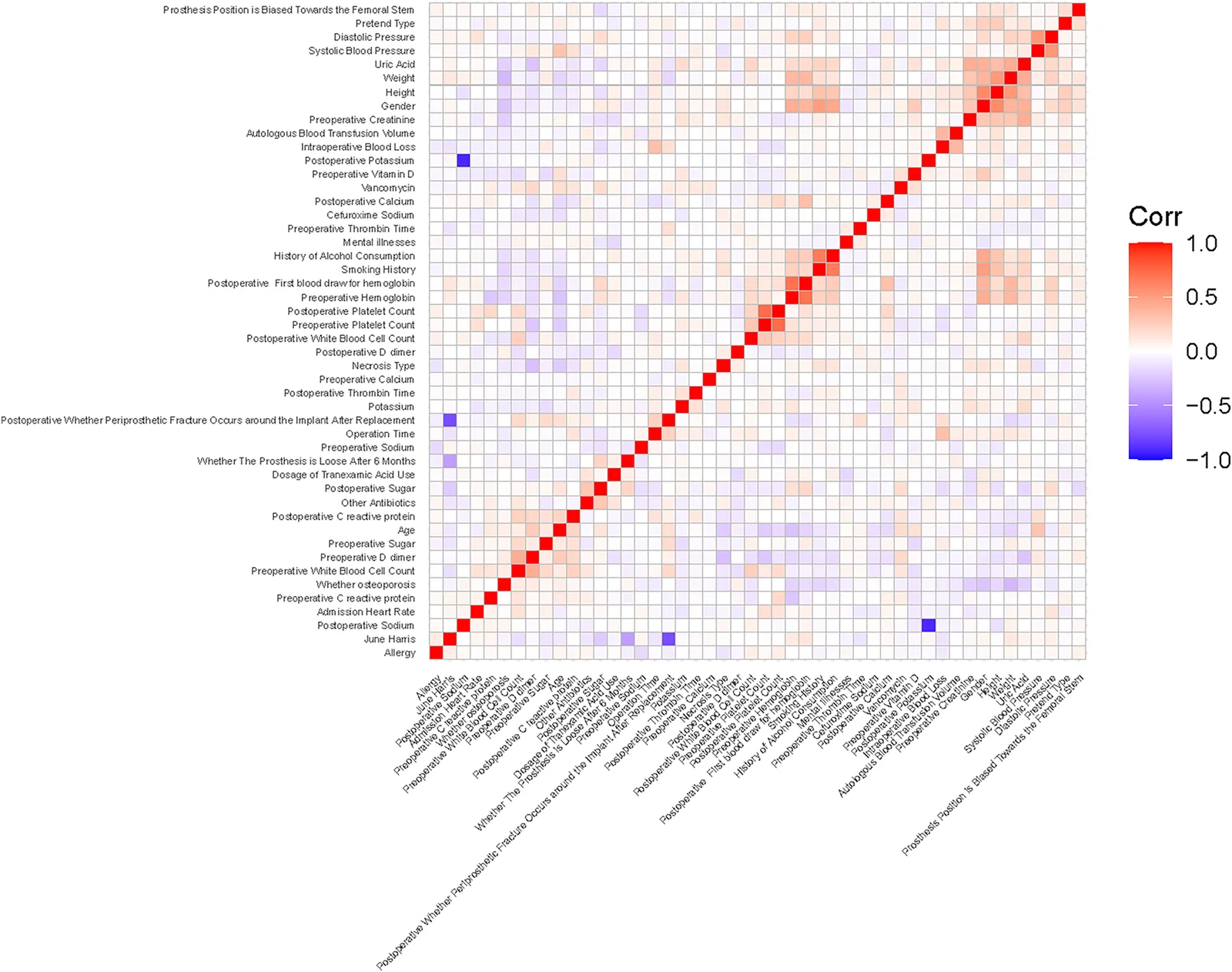
Pearson correlation matrices thermodynamic chart.

**Figure 4:**
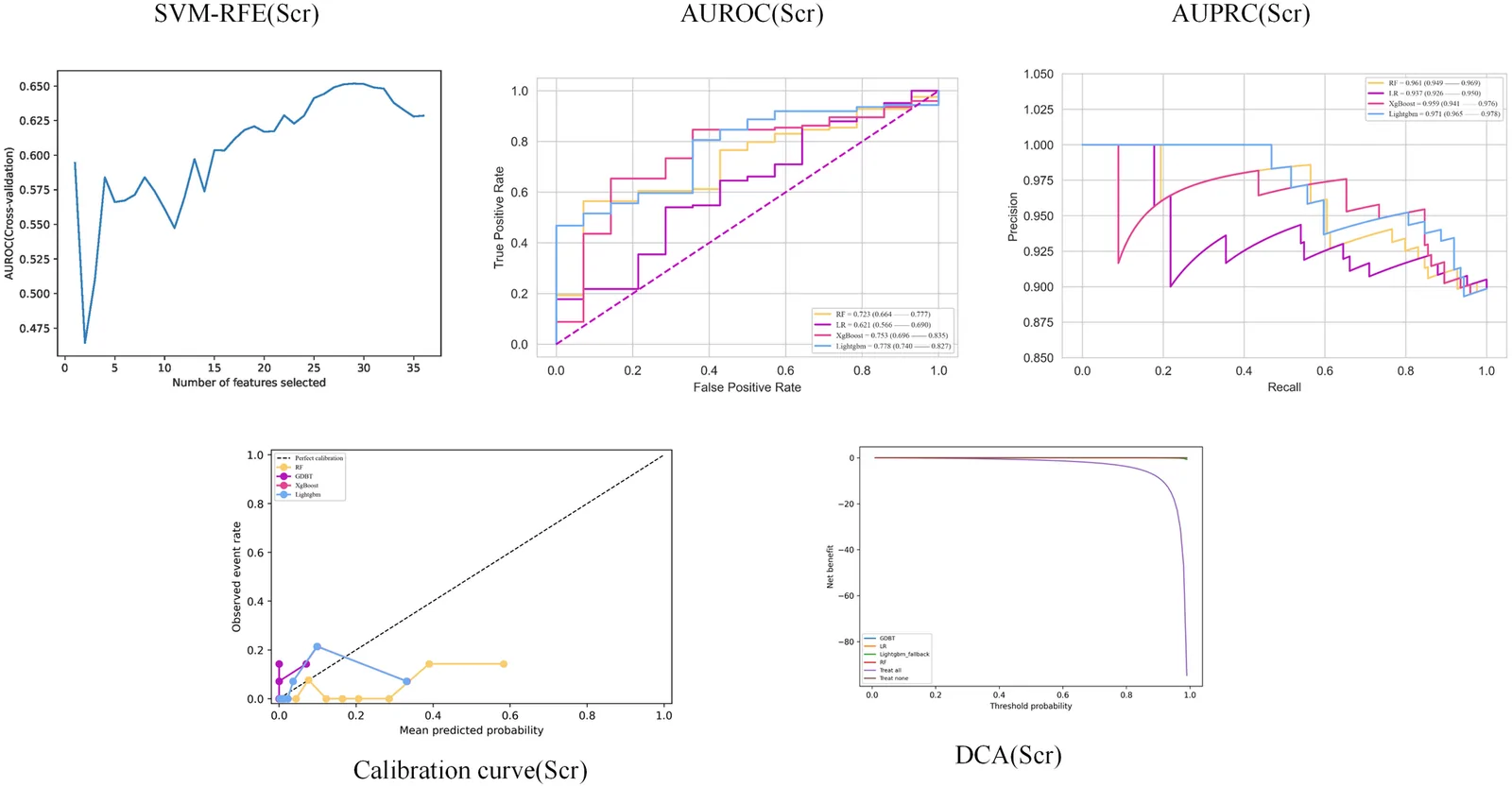
Model parameter diagram.

As shown in Figure 3, no pairwise correlations between continuous variables exceeded 0.8, suggesting no significant multicollinearity. Thus, all features were retained for subsequent feature selection. Using SVM-RFE, 28 predictors were identified as relevant for predicting Scr changes (Figure 4), including Gender, Age, Necrosis Type, Mental Illnesses, Smoking History, History of Alcohol Consumption, Admission Heart Rate, Height, Weight, Systolic Blood Pressure, Diastolic Pressure, Autologous Blood Transfusion Volume, Dosage of TXA, Vancomycin, Other Antibiotics, Procedure Type, Osteoporosis Status, Hemoglobin Difference, White Blood Cell Count Difference, Platelet Count Difference, Potassium Difference, Sodium Difference, Calcium Difference, CRP Difference, Thrombin Time Difference, D-dimer Difference, Preoperative Vitamin D, Presence of Periprosthetic Fracture, SUA, Fracture Around the Implant, and Prosthesis Position Biased Toward the Femoral Stem.

These selected features were used as input for four machine learning algorithms—GDBT, LR, RF, and XGBoost—to develop the prediction models. The optimal hyperparameters for each algorithm are summarized in Figure 1.

### Development and validation of prediction models

When evaluating the performance of the Scr change prediction model on the training cohort, the LightGBM model achieved an area under the curve (AUC) of 0.774 (95% CI: 0.720–0.829), higher AUC values for Scr predictions than the RF, GBDT, and XGBoost models, which achieved AUC values of 0.720 (95% CI: 0.657–0.774; DeLong test: *P* = 0.17), 0.619 (95% CI: 0.543–0.705; DeLong test: *P* = 0.08), and 0.748 (95% CI: 0.686–0.810; DeLong test: *P* = 0.64), respectively. Performance metrics on the validation set were consistent with those obtained from the training set.

The LightGBM model demonstrated superior predictive performance and robustness across both training and validation datasets. Based on these results, the LightGBM model was selected for further analysis of Scr changes. A summary of these results is provided in Table 3.

**Table 3:** Model performance evaluation (Scr).

Name	AUROC (95% CI)	Accuracy (95% CI)	AUPRC (95% CI)	F1 (95% CI)	Precision (95% CI)	Recall (95% CI)	Sensitivity (95% CI)	Specificity (95% CI)	DeLong Test *p* value
RF	0.720 (0.657–0.774)	0.820 (0.800–0.846)	0.960 (0.948- 0.970)	0.899 (0.886–0.914)	0.910 (0.899–0.921)	0.888 (0.866–0.913)	0.216 (0.119–0.307)	0.910 (0.899–0.921)	0.17
LR	0.619 (0.543–0.705)	0.639 (0.606–0.673)	0.937 (0.921- 0.954)	0.763 (0.738–0.788)	0.931 (0.915–0.954)	0.647 (0.610–0.678)	0.573 (0.475–0.713)	0.931 (0.915–0.954)	0.08
XgBoost	0.748 (0.686–0.810)	0.825 (0.802–0.852)	0.958 (0.94–0.973)	0.901 (0.887–0.917)	0.917 (0.906–0.930)	0.886 (0.863–0.913)	0.283 (0.188–0.406)	0.917 (0.906–0.930)	0.647
Lightgbm	0.774 (0.720–0.829)	0.885 (0.877–0.893)	0.970 (0.962–0.978)	0.939 (0.934–0.943)	0.897 (0.897–0.898)	0.984 (0.976–0.993)	0.000 (0.000–0.000)	0.897 (0.897–0.898)	

After replotting the calibration curve using a 10-interquintile interval, the observed event rate in the independent test set for serum creatinine outcomes was 3.62% (Figure 4). The Brier scores for the XGBoost, LightGBM, Gradient Boosting Decision Tree, and Random Forest models were 0.0380, 0.0381, 0.0429, and 0.0725, respectively, with average predicted probabilities of 5.35%, 5.33%, 0.72%, and 19.11%. Corresponding receiver operating characteristic curves and precision–recall curves for all models are shown in Figure 4. The decision curve analysis results demonstrate that our model exhibits positive net benefits across commonly used low-to-medium risk thresholds (see Figure 4).

### Model explainability

**Figure 5:**
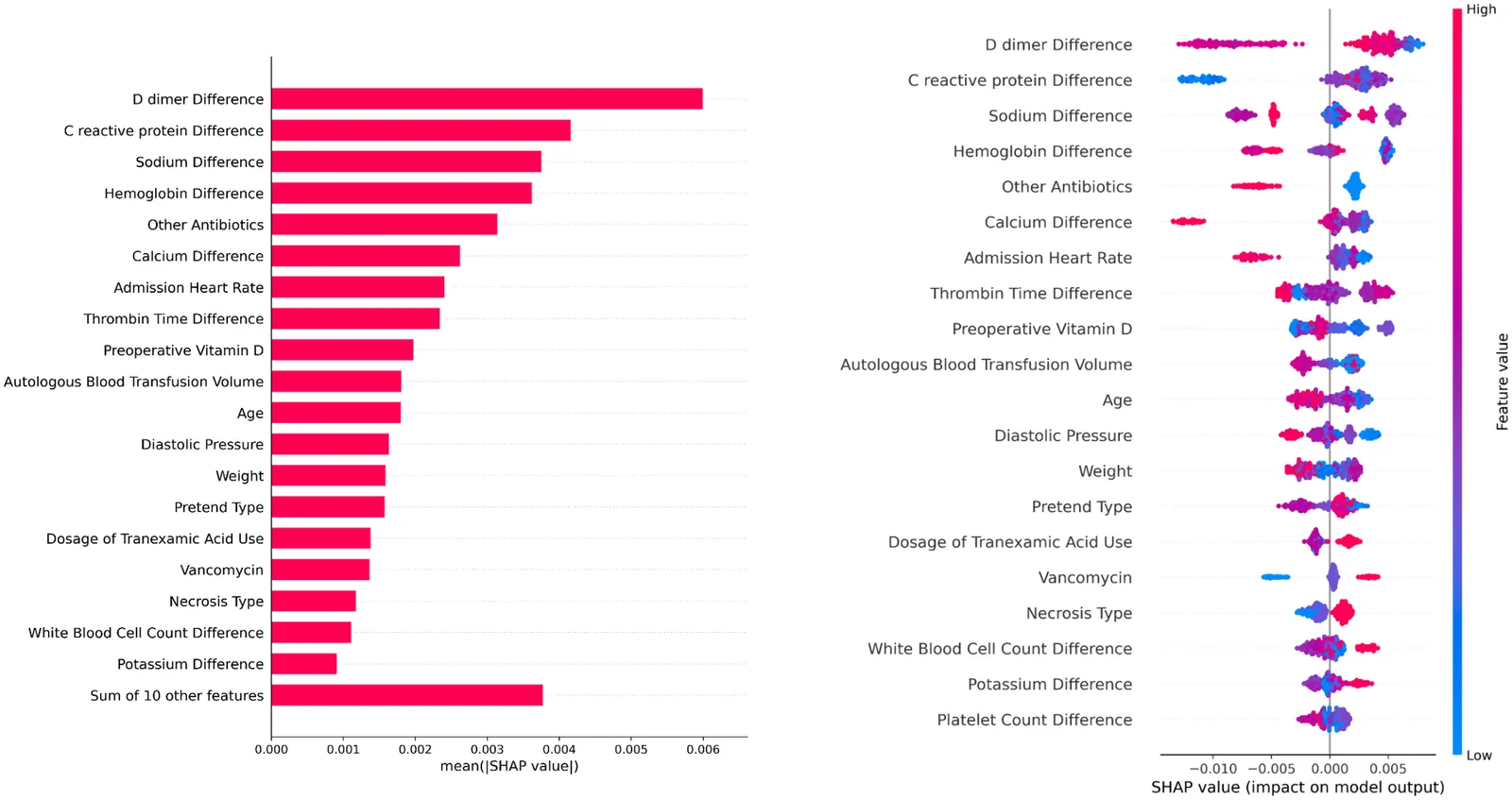
The SHAP summary plot (Scr).

The SHAP summary plot (Figure 5) displays the relative importance of the top 20 predictors in the LightGBM model for predicting Scr changes. The analysis identified D-dimer difference, CRP difference, sodium difference, hemoglobin difference, and the use of other antibiotics as the five most influential features.

**Figure 6:**
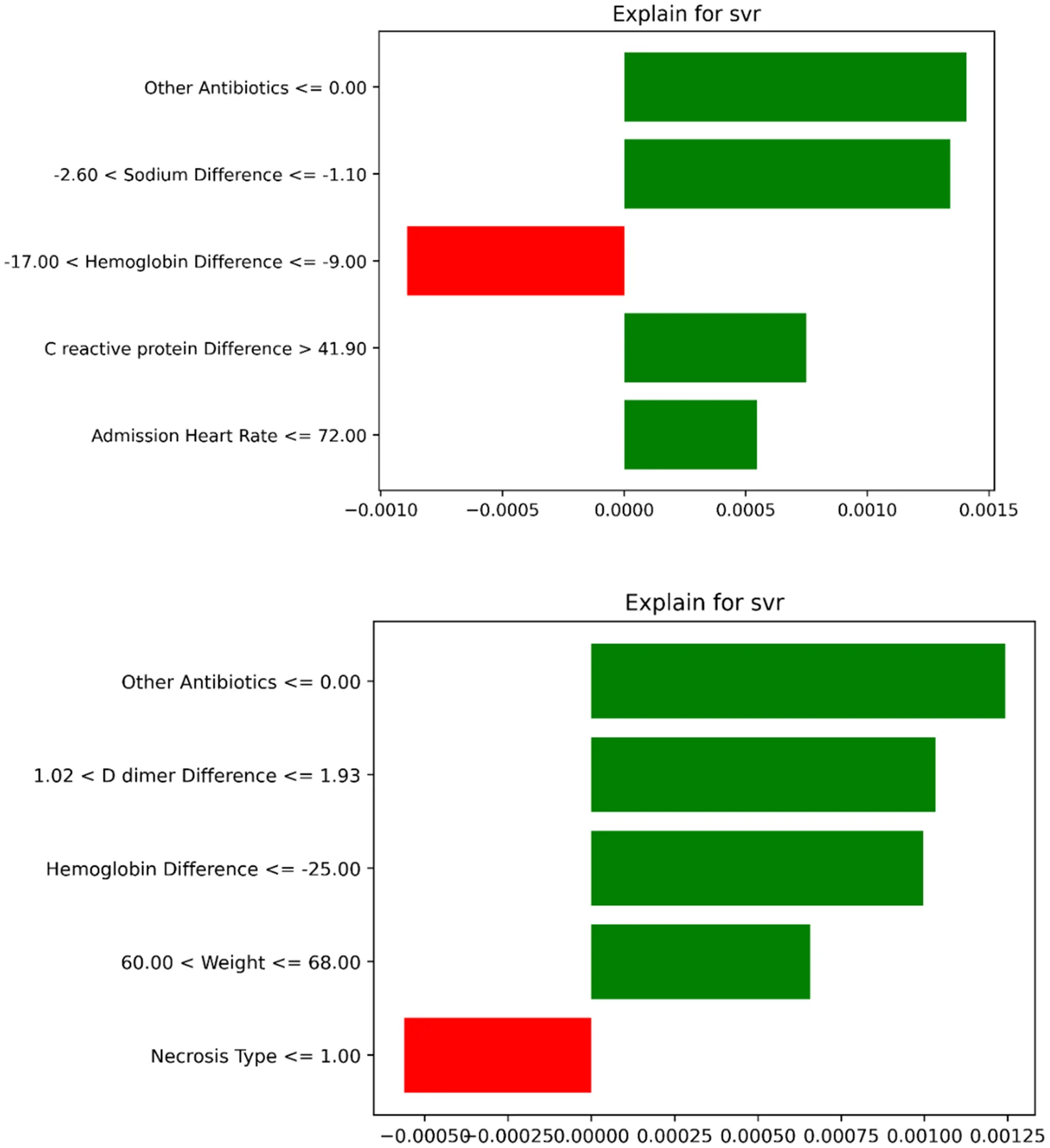
The Scr model is explained by a locally interpretable model. Features with green bars favor the results, while those with red bars contradict the results. X axle shows how much of each feature is added or subtracted from the patient's final probability value (i.e., a feature with a weight of 0.3 is equivalent to a 30% change in the probability of the outcome).

To interpret individual predictions, LIME was applied to representative instances from the LightGBM (Scr change). Feature contributions for each case are visualized in Figure 6, where green denotes features supporting the prediction and red indicates those contradicting it. In this case, an elevated Scr level was primarily driven by the use of other antibiotics, decreased serum sodium, greater intraoperative blood loss, and elevated D-dimer and CRP levels, whereas reduced blood loss exerted a protective effect.

## Discussion

Major orthopedic surgeries are typically associated with substantial tissue trauma and considerable blood loss, often necessitating the routine administration of hemostatic agents such as tranexamic acid (TXA) and prophylactic antibiotics during the perioperative period. TXA is widely used to minimize bleeding and transfusion requirements, while antibiotics are employed to prevent surgical site infections. However, this conventional therapeutic strategy may pose a potential risk of renal impairment: the antifibrinolytic action of TXA can compromise renal microcirculatory perfusion, and several antibiotic classes—including aminoglycosides and vancomycin—are known for their nephrotoxic properties (22–25). The concurrent use of these agents may lead to a synergistic nephrotoxic effect. Nevertheless, there remains a lack of systematic safety assessments based on real-world evidence regarding this interaction. Therefore, this study employs machine learning algorithms applied to real-world clinical data to develop predictive models for postoperative serum creatinine. Our objective is to comprehensively identify risk factors influencing renal function following major orthopedic procedures and to provide actionable insights for clinical decision-making. Shape-based feature importance analyses from the predictive models consistently indicate that intraoperative administration of tranexamic acid (TXA) has limited impact on postoperative serum creatinine (Scr) levels, whereas the dosage of antibiotics used during surgery exerts a discernible influence on Scr.

This finding is consistent with established clinical evidence: TXA, an antifibrinolytic agent, reduces surgical bleeding primarily through competitive inhibition of plasminogen activation. It does not exhibit direct nephrotoxicity, nor does it substantially affect the production or excretion of creatinine or uric acid. In contrast, many commonly used antibiotics carry well-documented nephrotoxic potential and are among the leading contributors to drug-induced kidney injury, particularly in high-risk perioperative settings. Thus, this study reinforces the notion that TXA, when used within standard dosing regimens, has minimal effect on key renal function biomarkers such as Scr and can be regarded as a safe and effective intervention for hemorrhage control. On the other hand, antibiotic dosing represents a modifiable risk factor for acute kidney injury (AKI). Judicious antibiotic selection, dose adjustment based on renal function, and close monitoring of renal parameters—including Scr trends and urine output—are essential strategies for mitigating surgery-associated AKI.

models also indicated that elevated serum C-reactive protein (CRP) levels exert a substantial influence on both serum creatinine (Scr). This finding is supported by well-established physiological and pathophysiological mechanisms and has been consistently observed across various clinical contexts (26, 27), including infection, autoimmune disorders, trauma, postoperative states, and gout flares. The effect of systemic inflammation, reflected by elevated CRP, on Scr involves the following mechanisms: (1) Hemodynamic Changes: Pro-inflammatory cytokines such as TNF-α, IL-1, and IL-6 promote renal vasoconstriction, diminish renal plasma flow, and reduce the glomerular filtration rate (GFR), thereby elevating Scr. (2) Direct Tubular Toxicity: Inflammatory mediators and reactive oxygen species generated during systemic inflammation can cause direct damage to renal tubular epithelial cells. (3) Microvascular Thrombosis: Severe inflammatory states activate coagulation pathways, potentially resulting in intrarenal microthrombi and impaired renal perfusion. (4) Secondary Systemic Complications: Inflammatory responses are frequently accompanied by hypotension, septic shock, or rhabdomyolysis, all of which represent additional insults predisposing to acute kidney injury (28–30).

Further analysis indicates that alterations in serum sodium significantly affect Scr (31). Mechanistically, postoperative sodium imbalances—whether hyponatremia or hypernatremia—often reflect underlying disturbances in water-electrolyte homeostasis, effective circulating volume, and osmotic regulation. These imbalances influence Scr through the following pathways: (1) Reduction in Glomerular Filtration Rate (GFR): A decline in GFR is a primary mechanism that elevates Scr, and it also contributes to reduced uric acid excretion. (2) Alterations in Renal Tubular Urate Transport: Volume depletion, which is frequently associated with hyponatremia or dehydration-related hypernatremia, enhances uric acid reabsorption and may suppress uric acid secretion, serving as a key mechanism that elevates Scr. (3) Acute Kidney Injury and Rhabdomyolysis: These conditions may develop secondary to significant electrolyte and volume imbalances, and can concurrently raise Scr.

Therefore, when elevated Scr is detected during postoperative monitoring, a thorough assessment of the patient's volume status and electrolyte balance—particularly sodium concentration—is warranted. Correction of volume and sodium disorders often constitutes a fundamental therapeutic strategy for improving renal function and uric acid metabolism.

Further analysis also identified intraoperative blood loss and transfusion volume as significant determinants of Scr levels (31). The underlying pathophysiological mechanisms include the following: (1) Activation of the Renin–Angiotensin–Aldosterone System (RAAS): Substantial hemorrhage triggers RAAS activation, leading to renal vasoconstriction and a consequent decline in glomerular filtration rate (GFR). (2) Enhanced Uric Acid Production: Perioperative hemorrhage-induced tissue hypoxia activates xanthine oxidase, accelerating the conversion of hypoxanthine to xanthine and ultimately to uric acid. (3) Transfusion-Related Oxidative Stress: Hemolysis following red blood cell transfusion releases free hemoglobin. When this exceeds the binding capacity of haptoglobin, it decomposes into heme and iron ions, promoting reactive oxygen species generation via the Fenton reaction and inducing renal tubular injury. (4) Transfusion-Associated Inflammation: Inflammatory mediators accumulate in stored blood products over time and may exacerbate systemic inflammation, further contributing to elevations in Scr. Collectively, these pathways indicate that major blood loss and transfusion during surgery elevate Scr through a “triple-hit” mechanism comprising prerenal (hemodynamic), intrinsic (oxidative and inflammatory), and postrenal (obstructive) insults. Therefore, patients undergoing extensive orthopedic procedures with considerable intraoperative blood loss warrant close postoperative monitoring of renal function and uric acid levels.

Based on these predictors, the LightGBM model for predicting Scr change consistent discriminative performance and calibration across the training, internal validation cohorts. The selected predictors were both biologically interpretable and clinically actionable. These models may assist clinicians in identifying high-risk patients and facilitate early risk stratification and inform perioperative management.

## Conclusions

The LightGBM model achieved optimal performance in predicting serum creatinine (Scr) changes. SHAP and LIME interpretability analyses indicated that tranexamic acid (TXA) dosage was not identified as a top predictor of postoperative acute kidney injury in our models; however, antibiotic dosage was correlated with renal function outcomes. Critical risk factors identified for postoperative acute kidney injury included elevated C-reactive protein (CRP) levels, perioperative sodium imbalances, substantial intraoperative blood loss, and transfusion volume. These results can assist clinicians in the early identification of patients at high risk of renal dysfunction and support the implementation of targeted preventive strategies to reduce the incidence of postoperative acute kidney injury.

### Strengths

Our study possesses several notable strengths. First, the predictor variables used closely reflect parameters that are routinely assessed in clinical practice, enhancing the real-world applicability of our models. Second,The modeling procedure adhered to rigorous development and validation protocols, utilizing multiple algorithms to select the optimal predictive model. Furthermore, comprehensive evaluation metrics and advanced interpretability methods, including SHAP and LIME, were employed to enhance model transparency and support clinical translation of the results.

### Limitations

Nevertheless, this study has several limitations. First, the single-center nature of the dataset and the limited sample size may affect the generalizability of the models. Although the prediction models demonstrated acceptable performance, with the area under the ROC curve (AUC) reaching 77%, this indicates there is still room for improvement in predictive accuracy. This may be attributed to the inherent complexity and multifactorial nature of postoperative renal function changes. In addition, the retrospective study design and the potential for unmeasured confounders or missing data may have resulted in the omission of additional variables with predictive value. The current LIME cases serve solely as illustrative examples of model decision logic, and their generalizability requires validation using larger datasets. The fact that the antibiotic and TXA dosage parameters were retained may introduce confirmation bias.

## Data Availability

The original contributions presented in the study are included in the article/Supplementary Material, further inquiries can be directed to the corresponding author/s.
